# Juvenile elastic arteries after 28 years of renal replacement therapy in a patient with complete complement C4 deficiency

**DOI:** 10.1186/1471-2369-13-161

**Published:** 2012-12-02

**Authors:** Florian Knoll, Emanuel Zitt, Denis Intemann, Karl Lhotta

**Affiliations:** 1 Department of Nephrology and Dialysis, Feldkirch Academic Teaching Hospital, Carinagasse 47, Feldkirch, 6800, Austria; 2 Department of Internal Medicine, Academic Teaching Hospital Feldkirch, Feldkirch, Austria; 3 Vorarlberg Institute for Vascular Investigation and Treatment, Academic Teaching Hospital Feldkirch, Feldkirch, Austria

**Keywords:** Complement, Atherosclerosis, Arteriosclerosis, Renal replacement therapy

## Abstract

**Background:**

Complement activation products are present in atherosclerotic plaques. Recently, binding of complement to elastin and collagen in the aortic wall has been demonstrated, suggesting a role of complement in the development aortic stiffness and atherosclerosis. The definitive role of complement in atherosclerosis and arteriosclerosis, however, remains unclear.

**Case presentation:**

We here describe a patient with hereditary complete deficiency of complement C4 suffering from Henoch-Schoenlein purpura and on renal replacement therapy for twenty-eight years. The patient had the full range of risk factors for vascular damage such as hypertension, volume overload, hyperphosphatemia and hyperparathyroidism. Despite that, his carotid artery intima media thickness was below the normal range and his pulse wave velocity was normal. In contrast, the patient’s coronary and peripheral muscular arteries were heavily calcified.

**Conclusion:**

This case supports the hypothesis that complement plays an important role in the development of stiffness of elastic arteries. We speculate that inability to activate complement by the classical or lectin pathways protected the patient from atherosclerosis, arteriosclerosis, stiffening and calcification of the aorta and carotid arteries. Inhibition of complement activation may be a potential target for prophylactic and therapeutic interventions.

## Background

Accelerated vascular calcification and vascular stiffening are common in end-stage renal disease (ESRD) [1-3]. Both are associated with mortality in the CKD and ESRD population [4]. No effective therapeutic strategy is available to prevent or slow down vascular calcification und arterial stiffening in patients with chronic kidney disease. It has recently been shown that complement components C3 and C4 bind to elastic and collagen fibers in the mouse aorta, and it was postulated that complement may play a central role in progressive vascular stiffening of elastic arteries [5]. We here describe a patient who, despite having multiple risk factors, does not show any signs of arteriosclerosis or atherosclerosis of his central elastic arteries after 28 years of renal replacement therapy. This unexpected finding is potentially related to the primary disease of the patient: hereditary complete deficiency of complement component C4.

## Case presentation

### Clinical history

The patient suffered from Henoch-Schoenlein purpura with severe renal involvement from the age of seventeen. From 1984 to 1985, aged 23 years, he underwent hemodialysis treatment. In 1985, he received his first renal allograft. Transplant function deteriorated because of interstitial fibrosis/tubular atrophy and recurrence of his primary disease. Immunofluorescence of the biopsies of the patient’s own kidneys and the transplant showed granular deposits of IgG, IgM, IgA and C3 in the mesangium and along the glomerular capillary walls. Staining for C4 was negative [6]. From 1989 to 1997 he again underwent hemodialysis. In 1996 a total parathyroidectomy with autotransplantation into the right brachioradial muscle was performed for severe hyperparathyroidism with a PTH of 820 pg/ml, hypercalcemia and hyperphosphatemia. After surgery PTH levels remained between 50 and 100 pg/ml. In 1997, a second renal transplantation was performed. In 2006, allograft function deteriorated and a transplant biopsy revealed advanced calcineurin inhibitor-associated arteriolopathy. Hyperphosphatemia despite treatment with various phosphate binders and a tendency to hypercalcemia were present during the years with reduced transplant function since 2006. Despite advanced renal failure, marked hypertension due to extensive fluid overload and despite severe hyperphosphatemia the patient refused to initiate dialysis until July 2011. Table 1 shows laboratory values and medications at commencement of dialysis.

**Table 1:** Laboratory values and medication before the patient started hemodialysis 2011

**Parameter**	**Value**	**Medication**	**Daily dose**
Creatinine	8.1 mg/dl	Sodium bicarbonate	1 g
Urea	310 mg/dl	Calcium carbonate	3 g
Calcium	2.42 mmol/l	Lanthanum carbonate	2250 mg
Phosphate	2.55 mmol/l	Sevelamer carbonate	7.2 g
PTH	103 pg/ml	Furosemide	500 mg
Haemoglobin	104 g/l	Moxonidine	0.2 mg
Triglycerides	93 mg/dl	Metoprolol	95 mg
Cholesterol	157 mg/dl	Amlodipine	10 mg
HDL-cholesterol	81 mg/dl	Darbepoietin	40 μg once weekly
LDL	72 mg/dl		

### Investigation of arterial vessels

**Figure 1:**
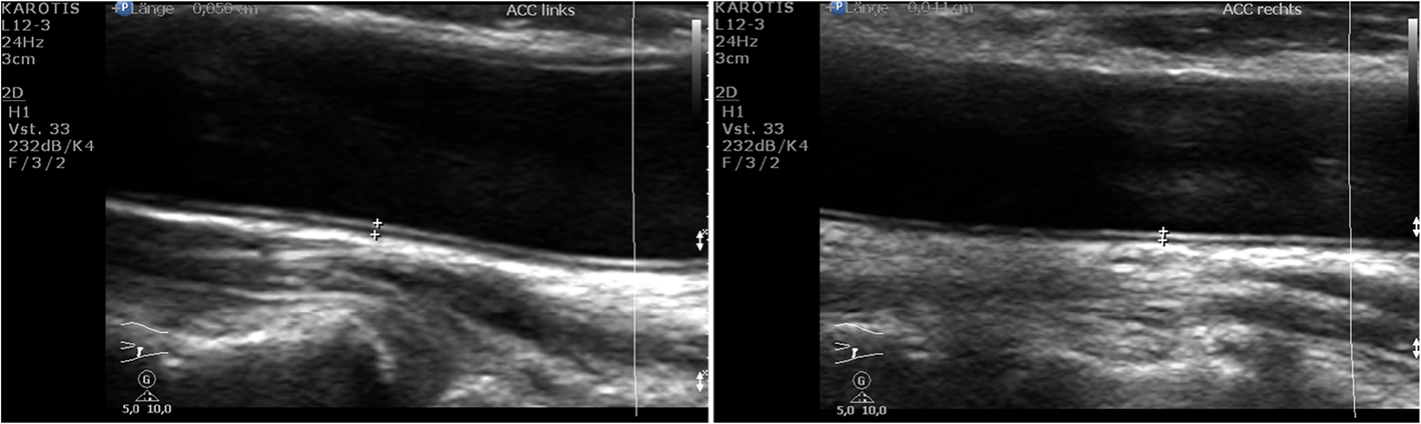
**B-mode carotid duplex ultrasound.** Right (right picture) and left (left picture) common carotid artery (CCA): no plaques and a normal carotid intima media thickness of 0.44 mm for the right CCA and 0.50 mm for the left CCA (upper limit of normal for age 50–59 years: 0.71 mm) as evidence of the absence of atherosclerosis and arteriosclerosis.

**Figure 2:**
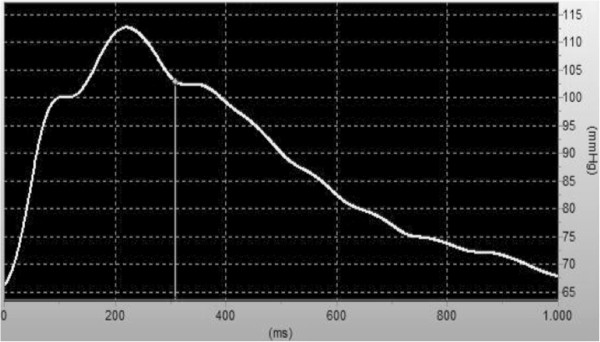
**Central aortic pulse wave.** No signs of arterial stiffness as indicated by low central pulse pressure augmentation. Brachial blood pressure was 125/65 mmHg, pulse pressure 60 mmHg; central aortic systolic/diastolic blood pressure 112 ± 1/66 ± 0 mmHg, central aortic pulse pressure 46 ± 1 mmHg; aortic augmentation pressure 11.5 ± 1.5 mmHg, aortic augmentation index 25 ± 2%.

At age 51 the patient underwent ultrasound of the carotid arteries as part of pre-transplant evaluation for a third kidney transplantation. Figure 1 shows the structure of the wall of both common carotid arteries to be completely normal without atherosclerotic plaques. The carotid artery intima-media thickness (CIMT) was 0.44 mm for the right common carotid artery (CCA) and 0.50 mm for the left CCA, thus on both sides clearly below the upper limit of 0.71 mm for a healthy population of the same age [7]. In addition, we assessed aortic augmentation index (Figure 2) and carotid-femoral pulse-wave velocity (PWV) using the Sphygmocor™ system (AtCor Medical, Sydney, Australia). The patient’s PWV of 5.3 ± 0.1 m/s is expected in a healthy adolescent, but is highly unusual in a patient who has been on renal replacement therapy for almost three decades. As a mean PWV a reference value of 11.6 ± 3.3 m/s has been proposed for patients with CKD 5 [8]. We did not detect signs of aortic calcification in thorax x-rays and in ultrasound investigations of the abdominal aorta and no signs of valvular calcifications in an echocardiography.

**Figure 3:**
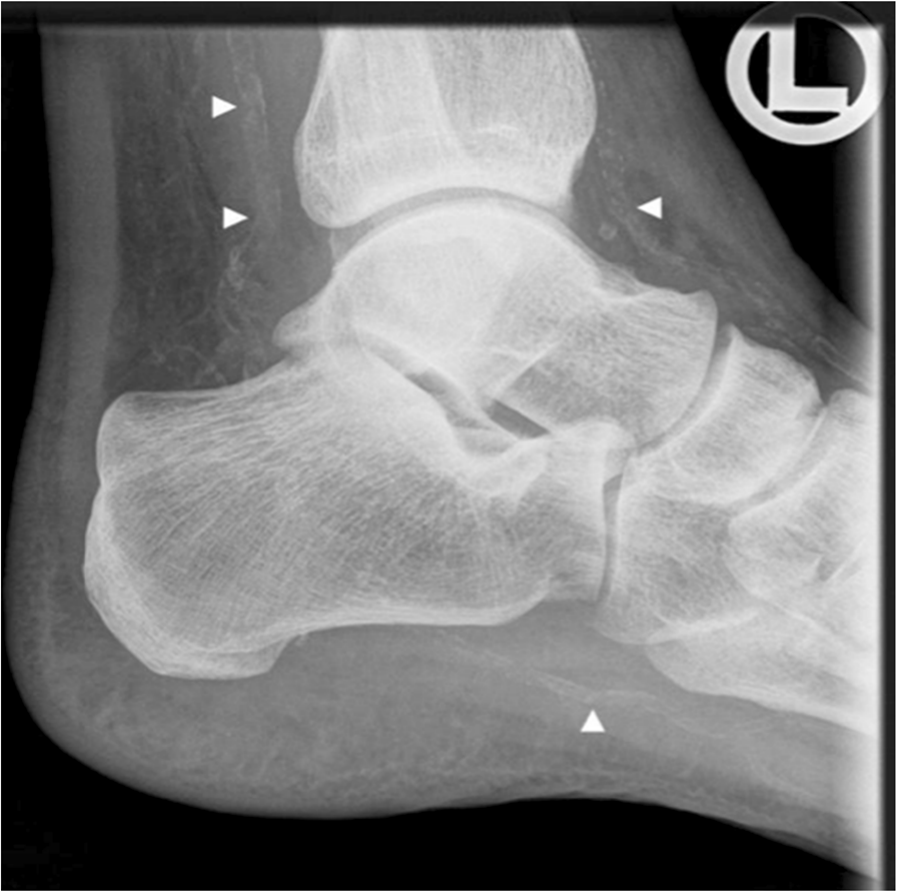
**X-ray of right ankle.** Severe calcification of the anterior and posterior tibial and plantar arteries is evident (white arrows).

**Figure 4:**
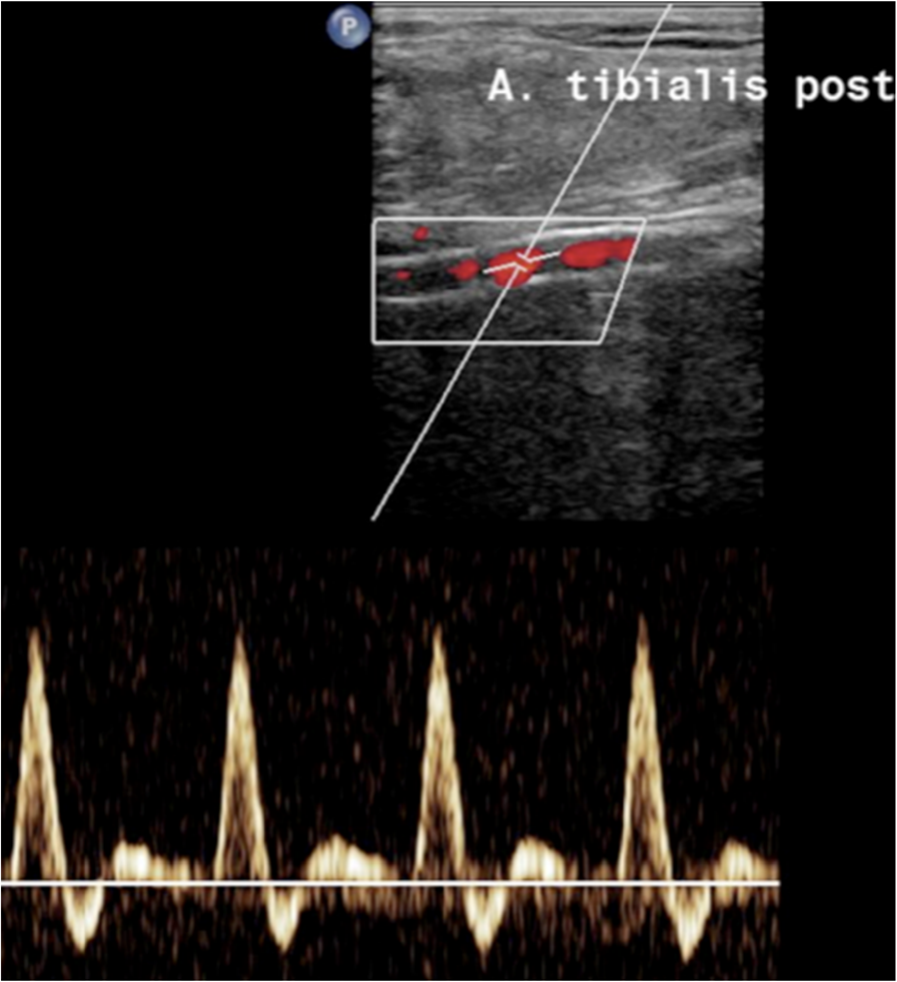
Duplex ultrasound and PW-Doppler sonography of right posterior tibial artery: severe calcification of the vascular wall and normal non-stenotic flow signal.

X-ray examinations of the patient’s hands and right foot (Figure 3) for joint pain revealed advanced calcification of the anterior and posterior tibial and plantar arteries and the radial artery, a finding to be expected in a patient with long-lasting kidney disease. Duplex ultrasound examination of peripheral muscular arteries (Figure 4) confirmed the presence of abundant vessel wall calcifications. The arterial blood flow signal was normal without signs of vascular stenosis. Computed tomography of the coronary arteries revealed severe calcification with an Agatston score of 782.5 corresponding to the 99^th^ percentile of age and gender matched controls [9]. Co-illustrated slices of the aorta were free of calcification.

### Complement C4 deficiency

The patient’s primary disease is hereditary complete deficiency of complement C4, and C4 was never detected in his serum on multiple occasions. The human complement C4 genes are located on chromosome 6 in the major histocompatibility complex region. They encode for two highly polymorphic isoforms, C4A and C4B. Hereditary complete C4 deficiency is exceptionally rare with less than 30 patients having been reported. Most of them suffered from a lupus-like illness. Our patient is homozygous for the MHC haplotype HLA A30 B18 DR7. This haplotype does not contain C4A genes, but instead two mutant short C4B genes with a 8127 g → a point mutation at the donor site of the intron 28 splice junction, which abrogates correct RNA splicing [10]. Splicing according to a new potential donor site seven nucleotides downstream would lead to a stop codon in exon 29. These results and the absence of C4 from our patient’s serum strongly suggest that he was completely C4-deficient.

## Discussion

Arteriosclerosis of central and peripheral arteries is the hallmark of the vasculopathy of chronic kidney disease. In addition to traditional risk factors, CKD-specific factors such as electrolyte abnormalities seem to drive vascular pathology in these patients. Our patient with almost 30 years of renal replacement therapy does not fit into this scheme. As expected, his coronary and peripheral muscular arteries were heavily calcified. He had, however, well preserved central elastic arteries.

It is highly unusual to find completely normal carotid vessels without intimal hyperplasia or atherosclerotic plaques and without calcification of the media in a patient who has been on renal replacement therapy for so long, including ten years of hemodialysis. The finding of a PWV of 5.2 m/s corresponds to the PWV of a young adult without arteriosclerosis of the aorta and is also unexpected [11]. He had suffered from the whole array of conditions that are thought to cause the vasculopathy of chronic kidney disease. The efficacy of these mechanisms is clearly illustrated by the calcifications of coronary and peripheral vessels. We believe that the patient’s primary disease, complement C4 deficiency, can explain these unusual findings.

### C4 and atherosclerosis

How might C4 deficiency protect against atherosclerosis? C4 is a main component of the classical and lectin pathways of complement. Without doubt, complement plays a role in the development of atherosclerosis. Terminal complement complex C5b-9 is regularly detected in atherosclerotic lesions [12]. In animal models, C6 deficiency protects against plaque formation [13]. Classical pathway activation may occur by several mechanisms in atherosclerosis. C-reactive protein, also present in lesions, is a potent activator of the classical pathway [14]. In addition, activation can be triggered by immune complexes containing autoantibodies against oxidized LDL and heat shock proteins [15].

### C4 and arteriosclerosis

Even more intriguing is the total absence of arteriosclerosis of central elastic arteries in this patient with C4 deficiency. Recent studies in mice support a role of complement in arteriosclerosis of these vessels [5]. It has been shown that complement components C3 and C4 are deposited along collagen and elastin fibers in the external elastic lamina of the aorta. These complement components are most likely derived from the perivascular adipose tissue of the aorta. Complement deposition increases with age and parallels vascular stiffening. In atherosclerosis-prone ApE(−/−) mice complement deposition extends into the media and intimal plaques. How C3 and C4 are deposited along collagen and elastin remains unclear. Spontaneously hydrolyzed C3 and C4 may attach covalently to the fibers. Alternatively, fibromodulin and adiponectin, which bind to collagen fibers in vessel walls [16,17], also bind to C1q and activate the classical complement pathway [18,19], which then would lead to complement deposition. As C4 is the central component of the classical pathway, the findings in our patient with C4 deficiency would favor the second mechanism and suggest that complement deposition in the vessel wall is mediated by the classical or lectin pathway. How complement activation eventually leads to vascular stiffening and calcification, remains to be determined. Complement fragments may attract leucocytes to the vascular wall, where they may be activated by complement and secrete proinflammatory cytokines. Elevated levels of interleukin-6 and tumor necrosis factor-alpha in the circulation are associated with vascular calcification in patients with chronic kidney disease [20]. They are also associated with low levels of fetuin-a, a potent inhibitor of calcification [21]. Complement activation may create a local environment in the vessel wall that strongly favors calcification. In an animal model of diabetes collagen synthesis and ventricular stiffening in the heart have been shown to be dependent on mannose-binding lectin and activation of the lectin pathway [22]. Likewise, a complement C5a receptor antagonist prevented cellular infiltration, fibrosis and stiffening of the heart in DOCA-salt hypertensive rats [23]. In analogy, complement activation may also accelerate fibrosis and stiffening of the central elastic vessels. Recently C4d deposits in the adventitia have been found in 50% of patients undergoing surgery of the ascending aorta. Lack of C4d staining was associated with aortic dissection. Whether complement may have a protective role against aortic dissection remains speculative [24].

## Conclusion

Our observations – normal central arteries but advanced calcification of coronary and muscular arteries - also would imply that arteriosclerosis of central elastic and peripheral muscular arteries (including coronary arteries) are driven by separate mechanisms. Whereas arteriosclerosis seems to be complement-dependent in elastic arteries, the process might be complement-independent in muscular arteries. Clearly, further research is needed to unravel the role of the complement system in atherosclerosis and arteriosclerosis. In addition, inhibition of complement activation may be a potential target for prophylactic and therapeutic interventions.

## Consent

The patient gave written informed consent to publication of his case history and all figures.

## Competing interests

The authors declare that they have no competing interests.

## Authors’ contributions

All authors are involved in the clinical management of the patient. FK, EZ and KL contributed to drafting and writing of the manuscript. DI performed ultrasound studies. EZ did the pulse wave velocity investigation and performed ultrasound studies. All authors read and approved the final manuscript.

## Pre-publication history

The pre-publication history for this paper can be accessed here:

http://www.biomedcentral.com/1471-2369/13/161/prepub
